# Bioinformatic prediction of the stereoselectivity of modular polyketide synthase: an update of the sequence motifs in ketoreductase domain

**DOI:** 10.3762/bjoc.20.131

**Published:** 2024-07-02

**Authors:** Changjun Xiang, Shunyu Yao, Ruoyu Wang, Lihan Zhang

**Affiliations:** 1 Department of Chemistry, Fudan University, Shanghai 200433, China; 2 Key Laboratory of Precise Synthesis of Functional Molecules of Zhejiang Province, Department of Chemistry, School of Science and Research Center for Industries of the Future, Westlake University, Hangzhou 310030, China; 3 Institute of Natural Sciences, Westlake Institute for Advanced Study, Hangzhou 310024, China; 4 Westlake Laboratory of Life Sciences and Biomedicine, Hangzhou 310030, China

**Keywords:** bioinformatics, conserved motifs, ketoreductase, polyketide synthase, stereocontrol

## Abstract

Polyketides are a major class of natural products, including bioactive medicines such as erythromycin and rapamycin. They are often rich in stereocenters biosynthesized by the ketoreductase (KR) domain within the polyketide synthase (PKS) assembly line. Previous studies have identified conserved motifs in KR sequences that enable the bioinformatic prediction of product stereochemistry. However, the reliability and applicability of these prediction methods have not been thoroughly assessed. In this study, we conducted a comprehensive bioinformatic analysis of 1,762 KR sequences from *cis*-AT PKSs to reevaluate the residues involved in conferring stereoselectivity. Our findings indicate that the previously identified fingerprint motifs remain valid for KRs in β-modules from actinobacteria, but their reliability diminishes for KRs from other module types or taxonomic origins. Additionally, we have identified several new motifs that exhibit a strong correlation with the stereochemical outcomes of KRs. These updated fingerprint motifs for stereochemical prediction not only enhance our understanding of the enzymatic mechanisms governing stereocontrol but also facilitate accurate stereochemical prediction and genome mining of polyketides derived from modular *cis*-AT PKSs.

## Introduction

**Figure 1:**
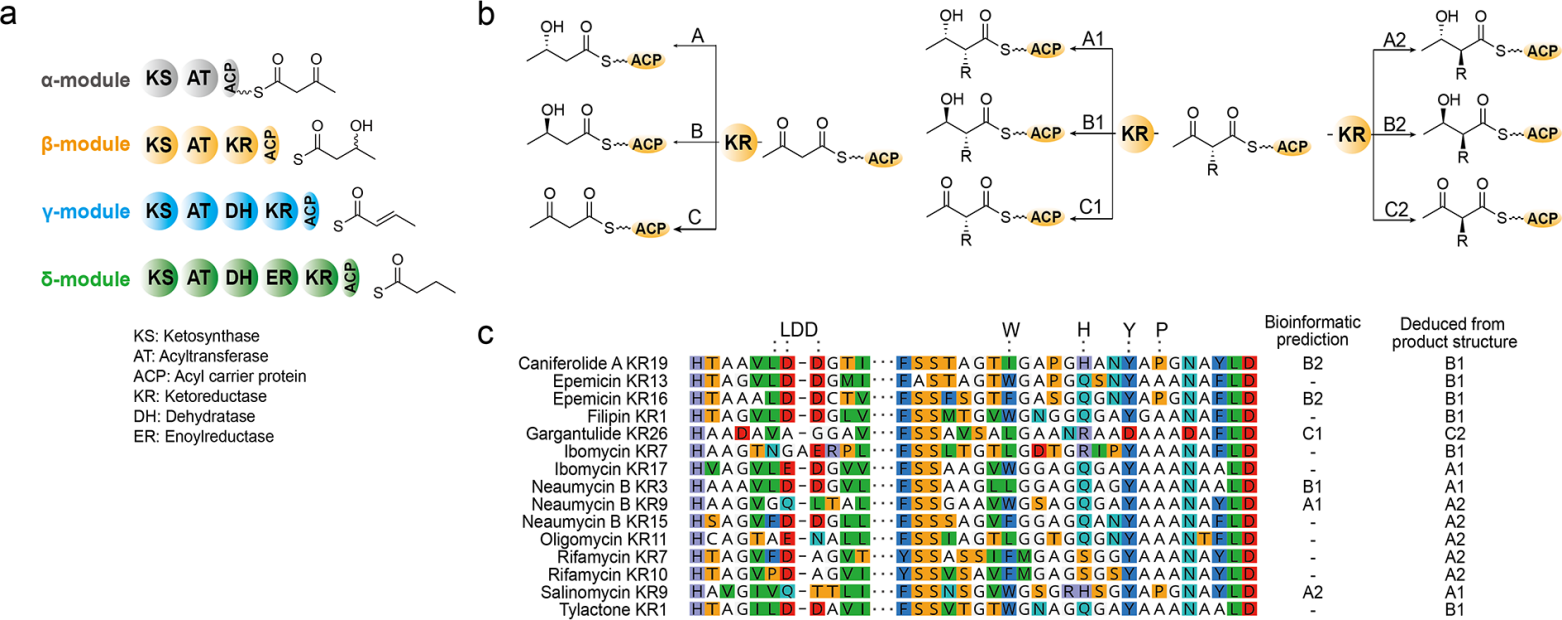
(a) Domain compositions and the products of α-, β-, γ- and δ-modules in *cis*-AT PKSs. (b) Stereocontrol of KR domains. KRs can be classified into A-type (β-ʟ-hydroxy), B-type (β-ᴅ-hydroxy), and C-type (reduction-incompetent; β-keto) depending on the product structure and into subtypes 1 (non-epimerizing, α-ᴅ-substitution product) and 2 (epimerizing, α-ʟ-substitution product). (c) Sequence alignment of KR domains whose stereoselectivity cannot be predicted based on the reported motifs. The labels show the product name and the order of the module where KR is located in the PKS assembly line (starting from the module with condensation function). KR types predicted based on the fingerprint motifs and deduced from product structure identified by chemical methods are shown on the right.

Type I modular polyketide synthases (PKSs) are large enzyme complexes that play a crucial role in the biosynthesis of bacterial polyketides, including many important clinical drugs such as erythromycin (antibiotic), epothilone (anticancer), ivermectin (antiparasitic), and spinosyn (insecticide) [1–2]. Modular PKSs consist of multiple modules that catalyze one round of chain extension by domains with different functions and are divided into *cis*-acyltransferase (*cis*-AT) and *trans*-AT PKSs depending on whether AT domain is embedded in the assembly line or not. All *cis*-AT PKS modules contain a ketosynthase (KS), an acyltransferase (AT), and an acyl carrier protein (ACP) to produce β-keto-intermediates, and some modules contain additional β-processing domains such as ketoreductase (KR), dehydratase (DH), and enoylreductase (ER) [3–4]. The building blocks for PKS biosynthesis often include malonyl-CoA or methylmalonyl-CoA, which are loaded onto the ACP by the AT domain. Subsequently, the KS domain catalyzes the decarboxylative Claisen condensation between the ACP-tethered extender unit and the KS-tethered growing chain. The elongated growing chain may undergo further processing by KR, DH, and ER domains, generating β-hydroxy, α,β-alkene, and saturated β-methylene groups, respectively (Figure 1a).

KR domains have garnered significant attention from researchers due to their ability to control the stereochemistry at the α*-* and β-positions of a polyketide chain [5–7]. KRs are classified into three types: A-type and B-type denote KRs that form ʟ- and ᴅ-configured β-hydroxy products, respectively, while C-type denotes KRs incapable of reducing the β-keto group [8–9]. KRs are further classified into subtypes 1 and 2 when the substrate contains an α-substition. Subtype 1 (A1, B1, and C1) KRs retain the original α-ᴅ-configuration, while subtype 2 (A2, B2, and C2) KRs epimerize the α-carbon to yield α-ʟ-configured products (Figure 1b). The stereocontrol of KR has been found to correlate with several conserved sequence motifs identified through bioinformatic and structural analyses (Figure 1c) [8–11]. A pioneering work by Caffrey identified that A-type KRs possess the conserved “W” motif but lack the “LDD” motif, whereas B-type KRs possess the “LDD” motif but lack the “W” motif, based on the sequence alignment of 68 KRs from 17 modular *cis*-AT PKSs [8]. In addition, Keatinge–Clay reported a conserved “H” motif in the sequence of A2-type KRs and a “P” motif in B2-type KRs as markers to distinguish them from the non-epimerizing A1/B1-type KRs [9]. The presence of the catalytic "Y" motif and the absence of the NADPH binding motif can be used to predict C2-type KRs [9]. These conserved motifs have been widely used to predict the stereochemical outcome of modular *cis*-AT PKSs and have facilitated bioinformatics-guided structural determination of complex polyketides [12–17].

However, despite being widely adopted, the prediction accuracy and the applicable range of these conserved motifs remain elusive, and not a few exceptions to these prediction rules have been reported (Figure 1c). For instance, the stereoselectivity of KR7 of ibomycin [12], KR15 of neaumycin B [14], and KR7 and KR10 of rifamycin [18] PKS cannot be accurately predicted due to the absence of both the LDD and W motifs. Moreover, KR3 of neaumycin B [14], KR19 of caniferolide A [15], KR16 of epemicin B [16], and KR26 of gargantulide B [17] show discrepancies between bioinformatic prediction and the determined product configuration. There are also instances where KRs contain or lack both of the LDD and W motifs, making it difficult to predict stereochemistry (Figure 1c).

With the increasing availability of whole genome sequencing, a growing number of PKS gene clusters and their products have been characterized [19]. To assess and evaluate the sequence–stereoselectivity relationship of KRs, here we have collected 1,762 KR sequences from modular *cis*-AT PKS gene clusters, whose product structures have been verified using spectroscopic and/or chemical methods. We reveal that the previously identified conserved motifs are best applicable to KRs in the β-module of actinobacterial PKSs but less applicable for other types of KRs. Moreover, we have identified additional fingerprint residues that improve stereochemical prediction. These fingerprint residues also suggest potential mode of interactions among KR, ACP, and DH domains, deepening our understanding of the stereocontrol of PKSs.

## Results and Discussion

### Preparation of KR sequence dataset

We first curated the amino acid sequences of KR domains from characterized bacterial *cis*-AT PKSs recorded in MIBiG database [20] and by manual literature review. In total, 1,762 KRs whose product structures were experimentally determined, such as by crystallography, nuclear magnetic resonance (NMR) analysis or by chemical synthesis, were obtained for further analysis.

The modules in PKSs were categorized as α-module (containing KS-AT-ACP tridomain), β-module (KS-AT-KR-ACP), γ-module (KS-AT-DH-KR-ACP), and δ-module (KS-AT-DH-ER-KR-ACP) based on their domain composition regardless of the domain activity [2] (Figure 1a). While the stereoselectivity of a KR in a β-module can be directly inferred from the product hydroxy group, the stereochemical outcome of a KR from a γ- and δ-module is obscured by the following dehydration by the DH domain. It is widely believed that an α,β-*trans* (*E*) double bond is generated from a ᴅ-β-hydroxy intermediate produced by a B-type KR, whereas a *cis* (*Z*) double bond is from an ʟ-β-hydroxy intermediate generated by an A-type KR via *syn* elimination [21]. However, based on the *syn-*elimination mechanism, the ʟ-α-methyl,ʟ-β-hydroxy substrate produced by A2-type KR can also result in a *trans* (*E*) double bond [18,22], and some DH domains are reported to have epimerase activity on the α-substitution [23]. Thus, the stereochemical outcomes of KRs in γ- and δ-modules cannot be directly inferred from the final product structure, and we discuss these KRs separately based on their module types.

**Figure 2:**
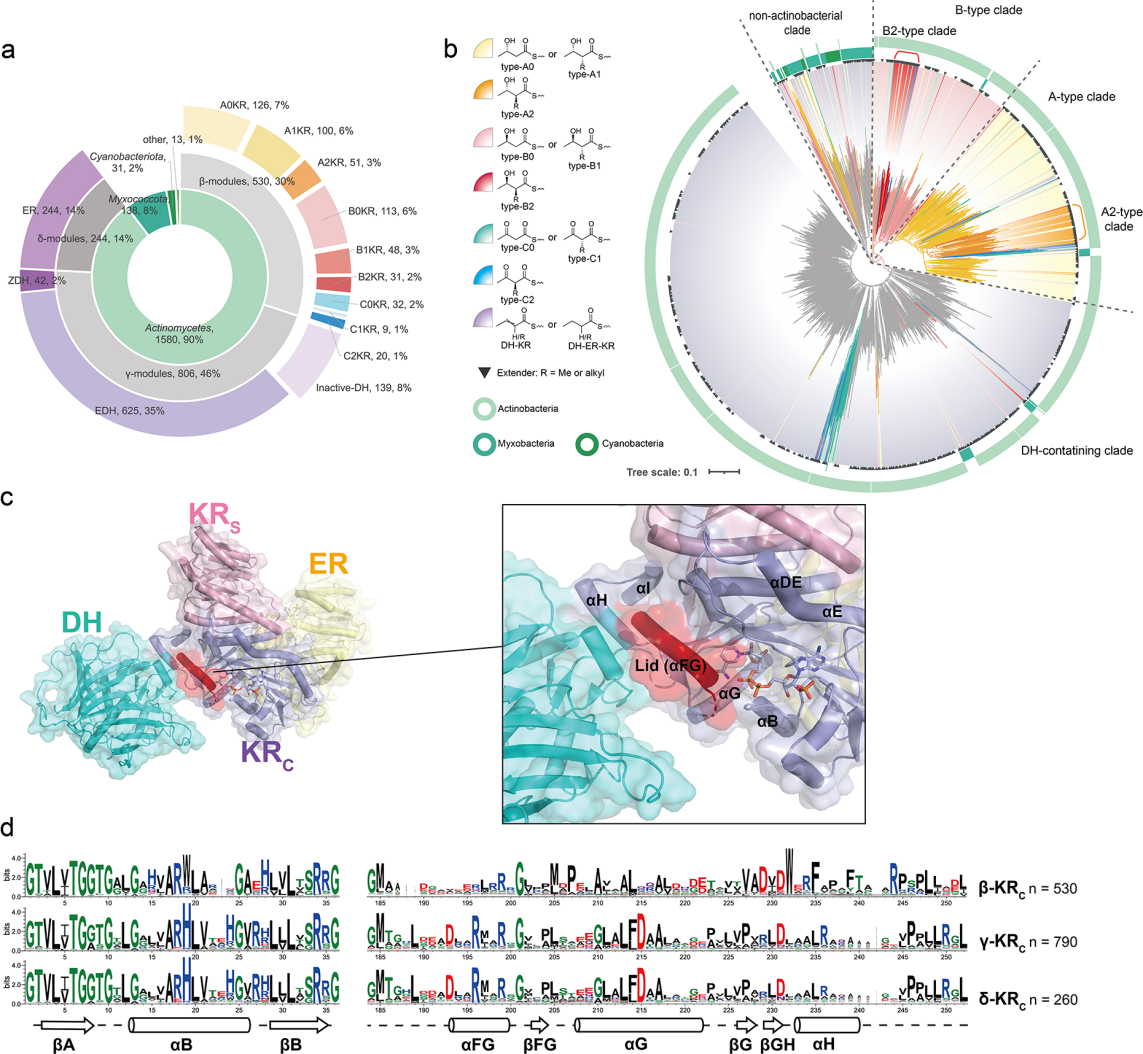
(a) Classification and distribution of the collected KR sequences according to host taxonomy, module types, and stereoconfigurations of the product. Here, “EDH” and “ZDH” respectively denote the *E*- (*trans*-) and *Z*- (*cis-*) configurations of a double bond. “ER” represents an α,β-alkane product. The numbers indicate the quantity of each KR type. (b) Phylogenetic analysis of the KR*_C_* subdomains. Product types were labeled by the inner color ranges, and the taxonomic origins were labeled by outer color rings. The triangles indicate a product having α-substituents. (c) Crystal structure of DH-ER-KR tridomain (PDB ID 8G7W) [24]. DH, KR*_S_*, ER, KR_C_ are colored in cyan, pink, yellow, and purple, respectively. The lid of KR catalytic pocket is colored in red. The co-crystallized cofactor is represented as sticks. (d) Sequence logo comparation of actinobacterial KR*_C_*s sorted by module types. The overall height of a stack in the logo indicates the level of conservation, while the width represents the number of amino acid residues at each position. For full-length KR*_C_* logos, see Figure S3 in Supporting Information File 1.

The curated 1,762 KR sequences were grouped into different types based on their product structure and the taxonomy of their host strains (Figure 2a). Among the KR sequences we collected, 90% are from *Actinomycetota*, and less than 10% are from *Myxococcota* and *Cyanobacteriota*. Among actinobacterial KRs, γ-modules accounted for more than half, of which approximately 78% resulting in a *trans* (*E*) double bond, 5% c*is* (*Z*) double bond, and the remaining 17% possessing an inactive DH domain. Among actinobacterial KRs from β-modules, A-type KRs (52%) are the most abundant, followed by B-type KRs (36%), and C-type KRs (12%).

### Comparison between KR*_S_* and KR*_C_* subdomains

KR domains are structurally divided into two subdomains: a catalytic subdomain (KR*_C_*) with an intact Rossmann fold where the active site resides, and a structural subdomain (KR*_S_*) with a truncated Rossmann fold that lacks NADPH binding sites and solely provides structural support by forming a heterodimer with KR*_C_* [24–26]. In δ-modules, an ER domain is inserted between KR*_S_* and KR*_C_*. Phylogenetic analyses of KR*_C_* and KR*_S_* revealed that both subdomains exhibited a cladogram pattern dependent on taxonomy, with cyanobacterial and myxobacterial KRs forming an ancestral clade followed by actinobacterial KRs (Figure 2b, and Supporting Information File 1, Figures S1 and S2). Such cladogram pattern was also observed in their KS domains [27], suggesting that they have diverged at an early point during evolution. Furthermore, both KR*_S_* and KR*_C_* trees showed that KRs belonging to γ- and δ-modules formed a separate clade distinct from those in β-modules, indicating that the existence of DH domain significantly influences the sequence of both KR subdomains [13]. This suggests a strong domain–domain interaction between KR and DH, as observed in the structure of KR-DH-ER tridomain (Figure 2c) [24], and indicates that KRs in β-modules and γ/δ-modules may not be generally interchangeable for domain swapping.

However, the phylogenetic cladograms of the two subdomains were significantly different within the clade formed by β-modules. In the KR*_C_* tree, stereoselectivity-dependent clades were formed, but such clades were not observed in the KR*_S_* tree (Figure 2b and Figure S1 in Supporting Information File 1). This indicates that the stereoselectivity of KR is controlled solely by KR*_C_*, and KR*_S_* does not influence its catalytic selectivity. Moreover, A2- and B2-type KR*_C_*s formed a separatable clade from A1- and B1-type KR*_C_*, respectively, suggesting that phylogenetic analysis can be used for stereochemical prediction. It is noteworthy that A0- (0 denotes as product without α-substitution) and A1-type KRs, and B0- and B1-type KRs cannot be distinguished in the phylogenetic trees of both subdomains. This implies that KRs likely exhibit promiscuity for α-substitution, and the structural differences in the α-position should not affect the original stereoselectivity of a KR. Such promiscuity can also be observed in the MycA KR in mycolactone biosynthesis, which accepts both α-substituted and α-unsubstituted substrates while retaining the same stereoselectivity for the β-hydroxy group [28].

To further investigate the sequence features of each module type, we performed sequence logo analyses of KR*_C_* subdomains from β-, γ- and δ-modules. Consistent with the phylogenetic cladogram, KR*_C_*s from γ- and δ-modules showed nearly identical logo features each other but were significantly different from the logo of β-modules. Major differences were observed in two regions of KR*_C_*, the *N*-terminal helix αB and the lid region αFG located at the *C*-terminal of KR*_C_* (Figure 2d). Indeed, this lid region exhibits a direct interaction with the DH and DH-KR linker (Figure 2c). However, such interaction was not observed between the *N*-terminal helix αB and DH in the crystal structure of DH-ER-KR tridomain (Figure 2c). This finding suggests that the helix αB may have an allosteric interaction with DH or potential large conformational changes between DH and KR during catalysis.

### Sequence logo analysis of KR*_C_* from β-modules

**Figure 3:**
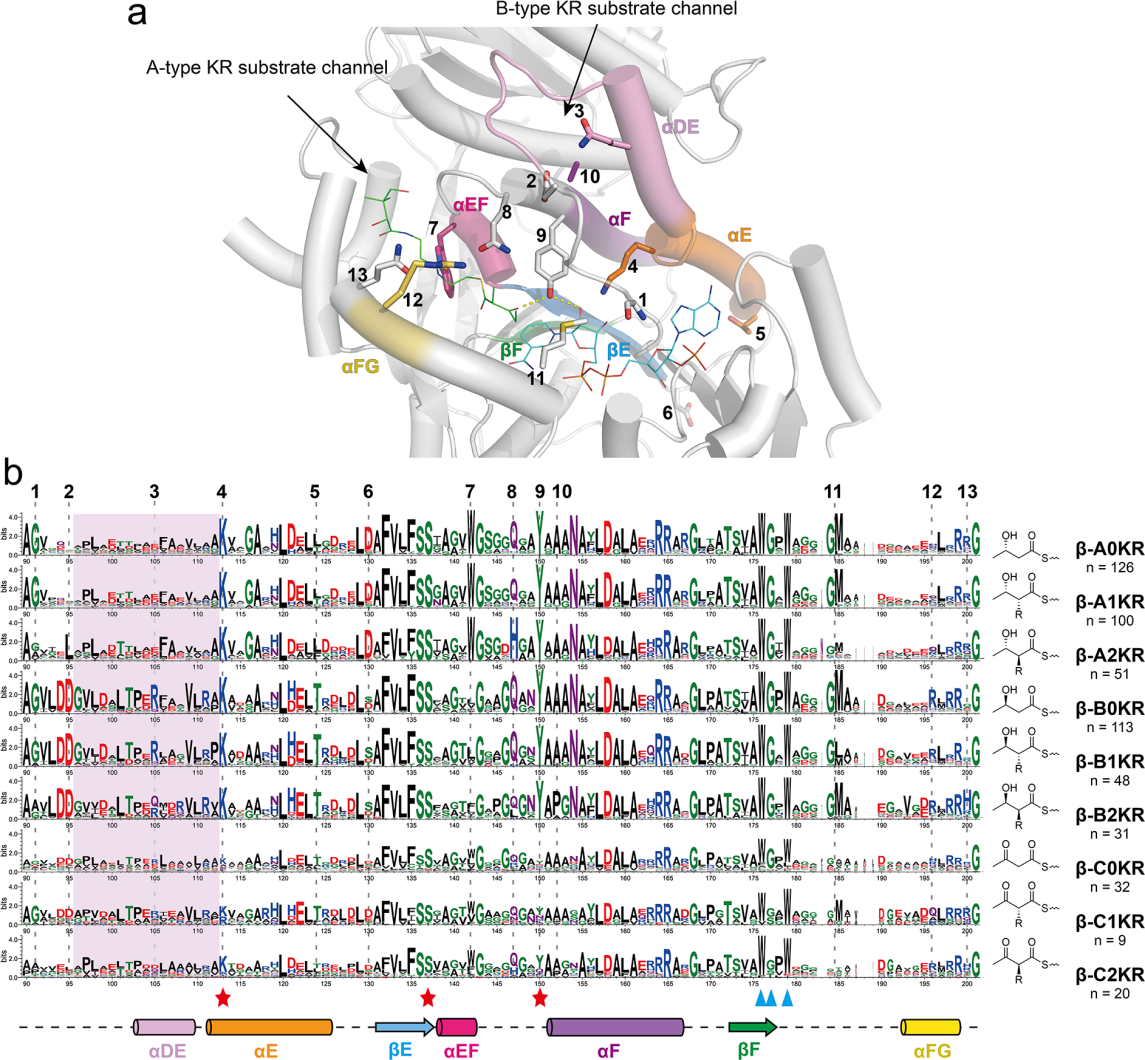
(a) The position of fingerprint motifs in the KR structure (AmpKR2, A1-type, PDB ID 5XWV) [29]. Fingerprints are shown as sticks. The co-crystallized substrate mimic and co-factor are shown as lines. (b) Sequence logo comparation of the core moiety of β-module KR*_C_* based on the classification of their products. The key catalytic residues are marked by red stars, and the NADPH-binding residues (partially) are marked by blue triangles. The numbers at the top indicate the fingerprint motifs.

Based on the clear stereoselectivity-dependent clades observed in the KR*_C_* tree of actinobacterial β-modules, we next carried out sequence logo analyses to identify key amino acid residues associated with stereoselectivity. Comparison of sequence logos among different types of KR*_C_*s revealed that the previously reported motifs [8–9], such as LDD (**2**) for B-type, W (**7**) for A-type, H (**8**) for A2-type, and P (**10**) for B2-type KR are highly conserved (Figure 3 and Figure S3 in Supporting Information File 1). In KRs from *Myxococcota* and *Cyanobacteriota*, while the LDD and W motif were mostly conserved, the H and P motifs characteristic for A2- and B2-type KRs, respectively, were not conserved (Figure S4). Thus, more characterized sequences are needed to better predict the stereochemistry at the α-position derived from non-actinobacterial KRs.

In actinobacterial KRs, we identified several new fingerprint motifs associated with stereoselectivity. First, A-type KRs and B-type KRs exhibited distinct variations around the αDE helix (highlighted in pink shadow area in Figure 3b). The difference is across the entire region, with the conserved R (**3**) in B-type KRs being most representative. This αDE helix region is located at the entrance of the predicted B-type KR substrate channel (Figure 3a) [9], suggesting its potential contribution for regulating substrate entry. On the αFG lid helix, a RLXR (**12**) motif was found in B-type KRs, while a XLXR motif was found in A-type KRs. A highly conserved T (**5**) was also identified in B-type KRs, whereas it was replaced by a more hydrophobic L in A-type KRs. On the loop between αE and βE, a conserved D (**6**) was found in A-type KRs.

For A2-type KRs, we identified a highly conserved L (**2**) in the position corresponding to the LDD motif (Figure 3). This residue may participate in interactions with the lid helix to adjust the pocket for epimerization of α-substituted intermediates. The lack of the conserved GM (**11**) motif preceding the αFG helix, which is highly conserved in other types of reductase-competent KRs, was also observed in A2-type KRs. The absence of this region in the electron density map of the A2-type AmpKR11 [30] may indicate a more flexible structure of the A2-type lid helix.

In B2-type KRs, we identified a conserved H (**13**) located at the end of lid helix (αFG) and the previously identified P (**10**) as signature motifs (Figure 3). Slight differences were observed in the loop region between αEF and αF, such as the more conserved P and less conserved Q, preceding the catalytic Y (**9**). Furthermore, some differences were noted in the αDE region downstream of the LDD motif, such as the R to Q mutation (**3**) and a conserved (D/E)RVLR between the R (**3**) and K (**4**) motifs, which may also regulate the structure of the loop forming the catalytic groove.

The sequence logo of C0/C1-type KRs showed that some of them possess the catalytic Y (**9**), but such KRs instead possess mutation in NADPH binding site (Figure S3 in Supporting Information File 1). In C2-type KRs, we found that most C2-type KRs possessed the catalytic Y (**9**), but some possessed Q at this residue. This substitution can be reasonable, as it serves as a hydrogen bond donor to activate the β-keto moiety of a substrate [31–32]. Additionally, the presence of K (**4**), which activates the catalytic tyrosine, was more conserved in C2-type KRs. In general, C2-KRs show similarity to either A2- or B2-KRs, but are likely to contain mutations in NADPH binding site [31].

### Sequence logo analysis of KR*_C_* from γ- and δ-modules

In the actinobacterial γ- and δ-modules, the stereochemical outcome of a KR is obscured by further dehydration catalyzed by DH. The dehydration mechanism by DH has been investigated in several DH domains, which all follow the *syn*-elimination mechanism [5,21,33]. Based on the product configuration, we classified DHs into two types, *E*-type DHs (*trans*-double bond) and *Z*-type DHs (*cis*-double bond) and analyzed sequence logos of the DH-associated KRs.

**Figure 4:**
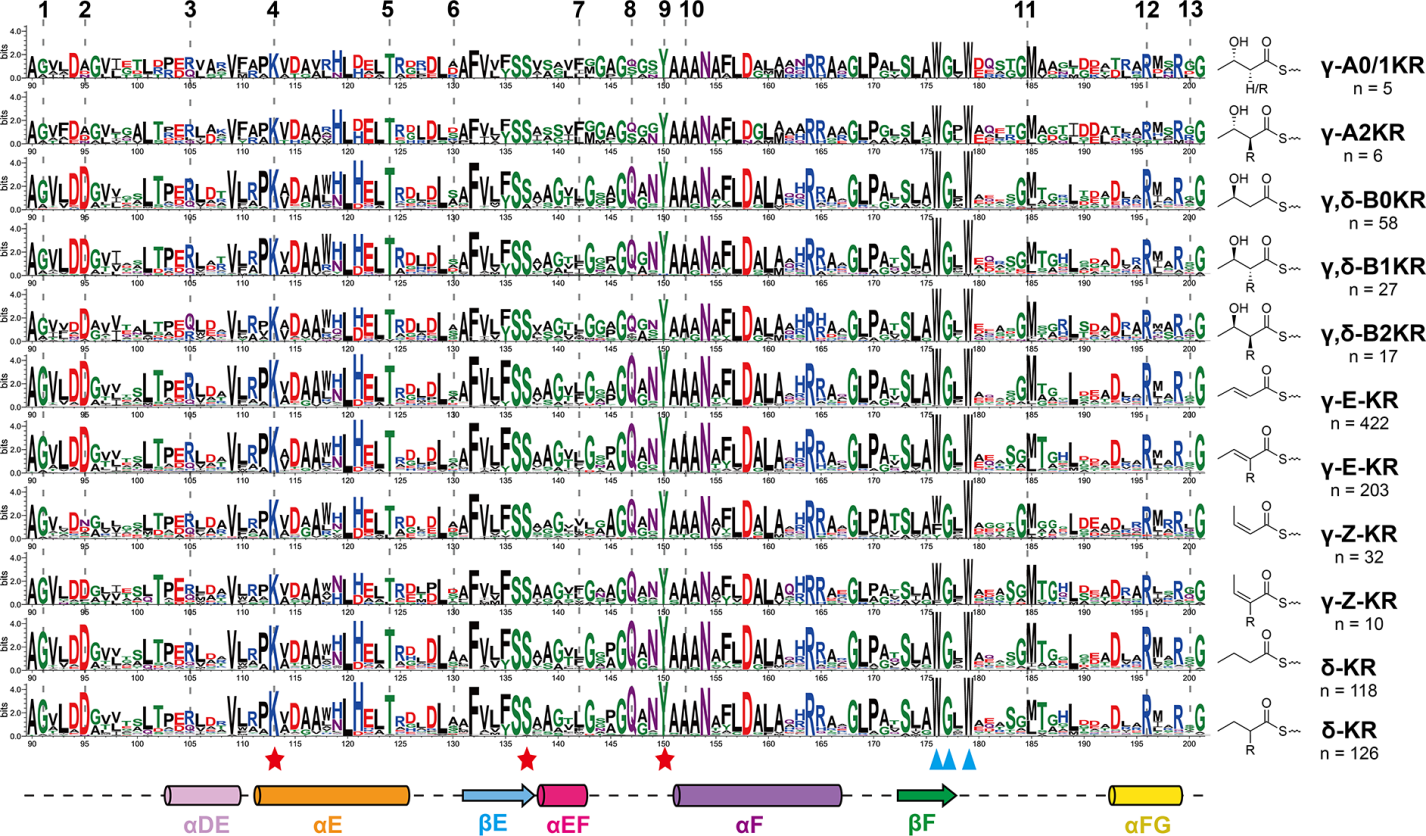
Sequence logo comparation of γ- and δ-module KR_C_ based on the classification of their products. Top five rows show KRs associated with an inactive DH that produces the hydroxy products. The key catalytic residues are marked by red stars, and the NADPH-binding residues (partially) are marked by blue triangles. The motif numbers at the top are corresponding to the location of fingerprints in Figure 3. For full-length sequence logo, see Figure S5 in Supporting Information File 1.

The sequence logo analysis of these KR*_C_*s revealed that the LDD (**2**) motif representative of B-type KR is highly conserved in KRs associated with *E*-type DHs (Figure 4 and Figure S5 in Supporting Information File 1), which is in agreement with previous proposal that the *trans* (*E*) double bond is formed from the ᴅ-β-hydroxyacyl intermediate produced by B-type KR, and a *cis* (*Z*) double bond is formed from the ʟ-β-hydroxyacyl intermediate [5]. However, only 5 out of 42 KRs associated with *Z*-type DHs contain the conserved W (**7**) motif found in A-type KRs. Additionally, some KRs associated with *Z*-type DHs possessed the LDD (**2**) motif. These observations suggest several possibilities: (1) the stereoselectivity prediction for KRs in γ- and δ-modules is inaccurate, (2) a *cis* (*Z*) double bond can also be produced from ᴅ-β-hydroxyacyl intermediates due to a different substrate binding mode in DH [7], or (3) a *cis* (*Z*) double bond is formed during post-PKS modification [22,34].

To gain a better understanding of the stereoselectivity of KRs in γ- and δ-modules, we focused on KRs associated with an inactive DH domain (DH^0^) that still produce a β-hydroxy intermediate. These KRs are phylogenetically mixed together with those in γ- and δ-modules with an active DH (Supporting Information File 1, Figure S2), making them good models for analyzing the stereoselectivity of DH-associated KRs. Based on product structures, we found eleven A-type KRs and as many as 102 B-type KRs from γ- and δ-modules, similar to the ratio of *Z*- and *E*-DHs (Figure 4). However, all A-type KRs associated with an inactive DH lacked the diagnostic W (**7**) motif, and other A-type characteristic motifs such as L (**5**) and D (**6**) were also missing or less conserved (Figure 4). Contrary, nearly all B-type KRs associated with an inactive DH possessed the second D in the LDD (**2**) and the T (**5**) motifs. B2-type KRs showed slightly less conserved LD in the LDD motif, which was also reported as a feature of KRs in *trans-*AT PKSs [11]. In γ-A2-type KR, the characteristic L (**2**) and H (**8**) motifs found in β-A2-type KR were absent, and γ- and δ-B2-type KR lacked the characteristic P (**10**) and H (**13**) features. Therefore, the situation for KRs in γ- and δ-modules is complicated: a KR containing the second D in the LD**D** motif is likely to be B-type, but the possibility of it being A-type cannot be excluded, and the stereochemistry of the α-carbon cannot be predicted.

### Towards better bioinformatic prediction of stereochemical outcomes

Based on the above analyses, here we provide an update in the scope and motifs used for bioinformatic prediction of the α-methyl (or -alkyl) and β-hydroxy stereochemistry in modular *cis-*AT PKSs. First, actinobacterial β-module KRs are good targets for bioinformatic stereochemical prediction by the previously reported motifs, and newly identified motifs can further support the prediction. However, other KRs cannot be predicted with high accuracy, especially for the stereochemistry at the α-position. Second, phylogenetic analysis of KR*_C_* subdomain is an effective means to predict stereoselectivity of KRs from actinobacterial β-modules. Specifically, our results indicate that the stereochemistry can be predicted well using the fingerprint motifs if the KR falls within the A- and B-type clades by phylogenetic analysis of KR*_C_* subdomain (and most of actinobacterial β-module KRs do fall within there). Overall, the motifs having correlation with stereoselectivity are summarized in Table S1 in Supporting Information File 1. Below we provide a detailed guideline for stereochemical prediction of KRs.

**Figure 5:**
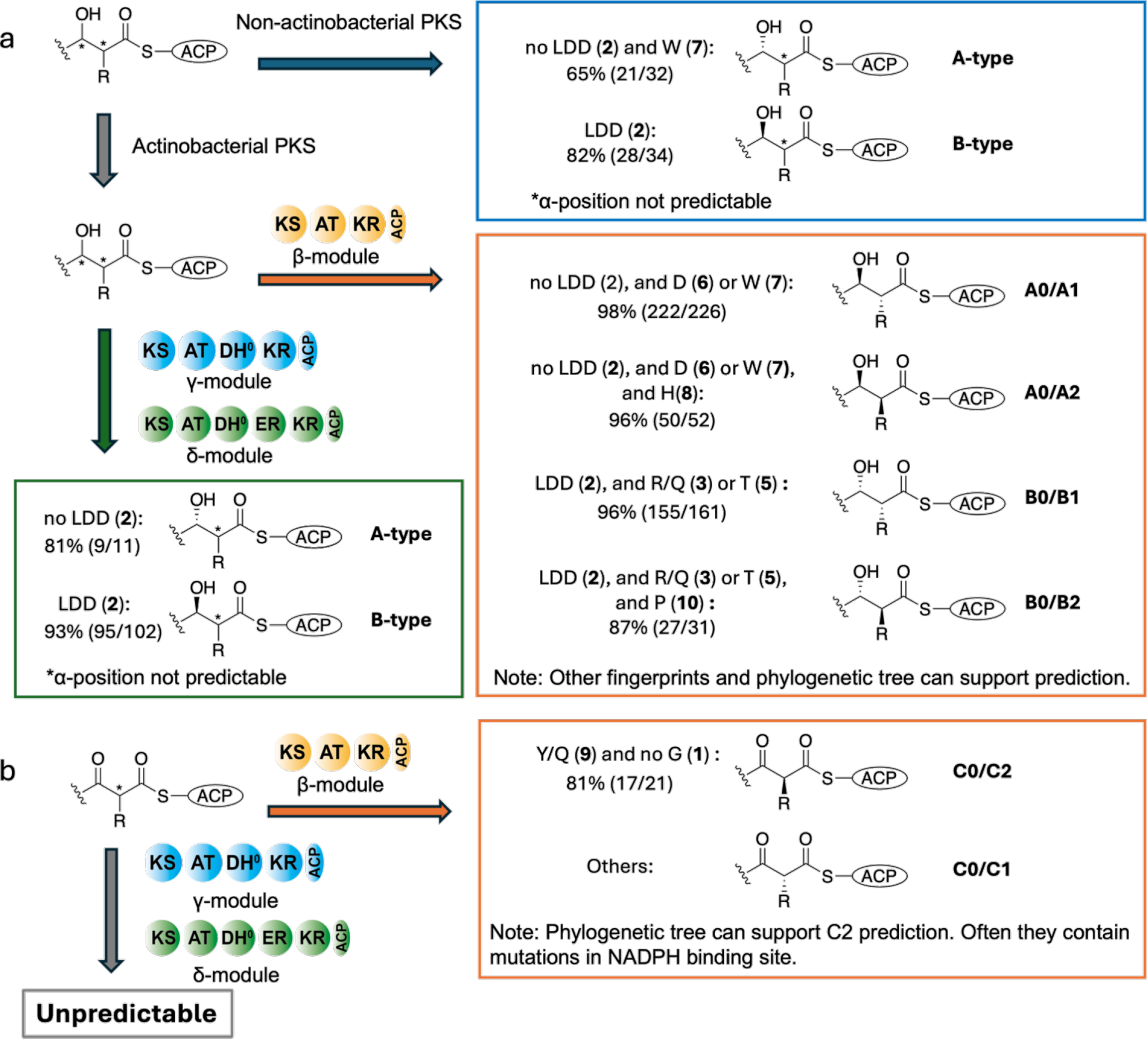
Summary of the updated fingerprints sorted by the taxonomic origin and the module type. Percentage numbers show KRs meeting the fingerprint description in our curated dataset. (a) Motifs useful for the stereochemical prediction of β-hydroxyl products. (b) Motifs useful for the stereochemical prediction of α-methyl,β-keto products.

For non-actinobacterial PKSs, stereochemistry can be predicted by the presence of LDD motif for B-type. The absence of the LDD and the presence of the W motif are indicative for A-type, but with moderate accuracy (Figure 5).

For actinobacterial β-module KRs, stereochemistry can be predicted by using the LDD and W, as well as the newly identified R/Q (3), T (5), and D (6) motifs as summarized in Figure 5. About 87% to 98% of the KRs belonging to each product type satisfied our prediction criteria, verifying the accuracy of stereochemical prediction within this group of KRs.

In contrast, for actinobacterial γ- and δ-module KRs, the newly identified motifs cannot guide the prediction, and only A- and B-type can be roughly predicted by the presence and absence of the LDD motif. However, phylogenetic analysis can support bioinformatic prediction if a KR falls within A1/A2 or B1/B2 clades: for example, fostriecin KR2, which produces a *cis* double bond together with a DH, locates in the A1-type clade, in agreement with its stereoselectivity [35].

Lastly, we applied our prediction criteria for the previously mis-predicted or unpredictable β-module KRs, for example due to having both LDD and W motifs, for validation. Among nine KRs we analyzed, five can be accurately predicted by either conserved motif analysis or by phylogenetic analysis, illustrating the advantage of using multiple strong predictive motifs (Table S2 in Supporting Information File 1). It is also worth noting that some of the KRs analyzed here may contain wrong stereochemical assignment of the product, potentially derived from misinterpretation of NMR analyses [14,36–37]. Nevertheless, many of the previously reported unpredictable KRs are from non-actinobacteria or from γ- and δ-module KRs, and bioinformatic prediction of these KRs would require further studies and more characterized sequences.

## Conclusion

In summary, we systematically assessed the stereoselectivity-associated sequence motifs from 1,762 KR sequences in bacterial modular *cis*-AT PKSs whose product structures were experimentally determined. Our analyses revealed that different KRs, by taxonomic origin or by module types, have differences in the fingerprint motifs, affecting the prediction accuracy by conserved motif analysis. We identified several additional fingerprint motifs in the KR*_C_* subdomain that can be used to better predict the stereochemistry of KRs from actinobacterial β-modules. The identified motifs reside at the interface of domain–domain or domain–substrate interactions, shedding light on the enzyme mechanism for stereocontrol. Our work provides an overview for current bioinformatic prediction of stereoselectivity of KR in *cis*-AT PKS, expands the understanding of the stereocontrol of PKS from bioinformatics perspective, and will facilitate accurate stereochemical prediction and genome mining of complex polyketides.

## Experimental

### Sequence collection and screening

All *cis*-AT PKS and PKS-NRPS amino acid sequences recorded in MIBiG, as well as the PKS sequences reported in literatures we have curated were targeted for further analysis. Sequences with multiple modules on a peptide chain were cut into single-module sequences based on the boundaries annotated of modules. These sequences were then filtered according to the following rules: (a) The PKS sequences that produce the same final product but originate from different strains of bacteria only retain the sequences from one of the sources. For example, only the erythromycin PKS from *Saccharopolyspora erythraea* were retained. (b) To associate sequence information with reliable absolute configuration of the product, the stereochemical configurations of collected polyketide products were manually checked by literature searches. Absolute configurations determined entirely by chemical methods, such as crystal structures, NMR, chemical degradation and derivatization, were considered reliable. Alternatively, relative configurations determined by NMR methods that corresponded exactly to the results predicted by bioinformatics were also considered reliable. Compounds of which only the relative configurations were elucidated were excluded from the dataset. (c) Sequences for which it was impossible to infer the stereochemistry of KR product were removed, such as M1 to M3 of rifamycin PKS whose product experiences later aromatization. (d) Modules with unconventional module compositions (e.g., modules with two KR domains), modules without KR domains, and loading modules were excluded from the data. After organizing and filtering, the KR*_S_* and KR*_C_* subdomain sequences were extracted for further bioinformatic analysis.

### Bioinformatic analysis

The multiple sequence alignment, sequence logo analysis, and tree building were performed on Geneious Prime 2023.2 (https://www.geneious.com) with options shown below: multiple sequence alignments were generated by MAFFT 7.450 (E-INS-i option), Neighbor-Joining trees were built by Geneious Tree Builder using Jukes-Cantor distance model. Sequence logo visualization was conducted by WebLogo 3. Protein structural analysis was conducted by PyMOL.

## Supporting Information

File 1 Supporting Figures and Tables.

File 2 Supporting dataset.

## Data Availability

All data that supports the findings of this study is available in the published article and/or the supporting information to this article.
