# Supplementary material for: Bioinformatic prediction of the stereoselectivity of modular polyketide synthase: an update of the sequence motifs in ketoreductase domain
Source: Beilstein J Org Chem. 2024 Jul 2;20:1476–85. doi: 10.3762/bjoc.20.131 (PMC11228615; doi:10.3762/bjoc.20.131)
Supplement: File 1 — Supporting Figures and Tables. [file Beilstein_J_Org_Chem-20-1476-s001.pdf]

BEILSTEIN JOURNAL OF ORGANIC CHEMISTRY

Supporting Information

for

Bioinformatic prediction of the stereoselectivity of modular polyketide synthase: an update of the sequence motifs in ketoreductase domain

Changjun Xiang, Shunyu Yao, Ruoyu Wang and Lihan Zhang

Beilstein J. Org. Chem. **2024,** *20,* 1476–1485. doi:10.3762/bjoc.20.131

Supporting Figures and Tables

License and Terms: This is a supporting information file under the terms of the Creative Commons Attribution License (https://creativecommons.org/licenses/by/4.0). Please note that the reuse, redistribution and reproduction in particular requires that the author(s) and source are credited and that individual graphics may be subject to special legal provisions.
The license is subject to the *Beilstein Journal of Organic Chemistry* terms and conditions: (https://www.beilstein-journals.org/bjoc/terms)

Table of contents

Figure S1. Phylogenetic tree of KR_S.

Figure S2. Phylogenetic tree of KR_C in a rectangular format.

Figure S3. Sequence logo comparation of actinobacterial KR_Cs

Figure S4. Sequence alignment of KR_Cs from non-actinobacterial *β*-modules

Figure S5. Sequence logo comparation of KR_C from actinobacterial γ- and δ-modules

Table S1. Stereospecificity-conferring motifs summary of KRs from actinobacterial *β*-modules.

Table S2. Stereochemical prediction of selected KR sequences.

References

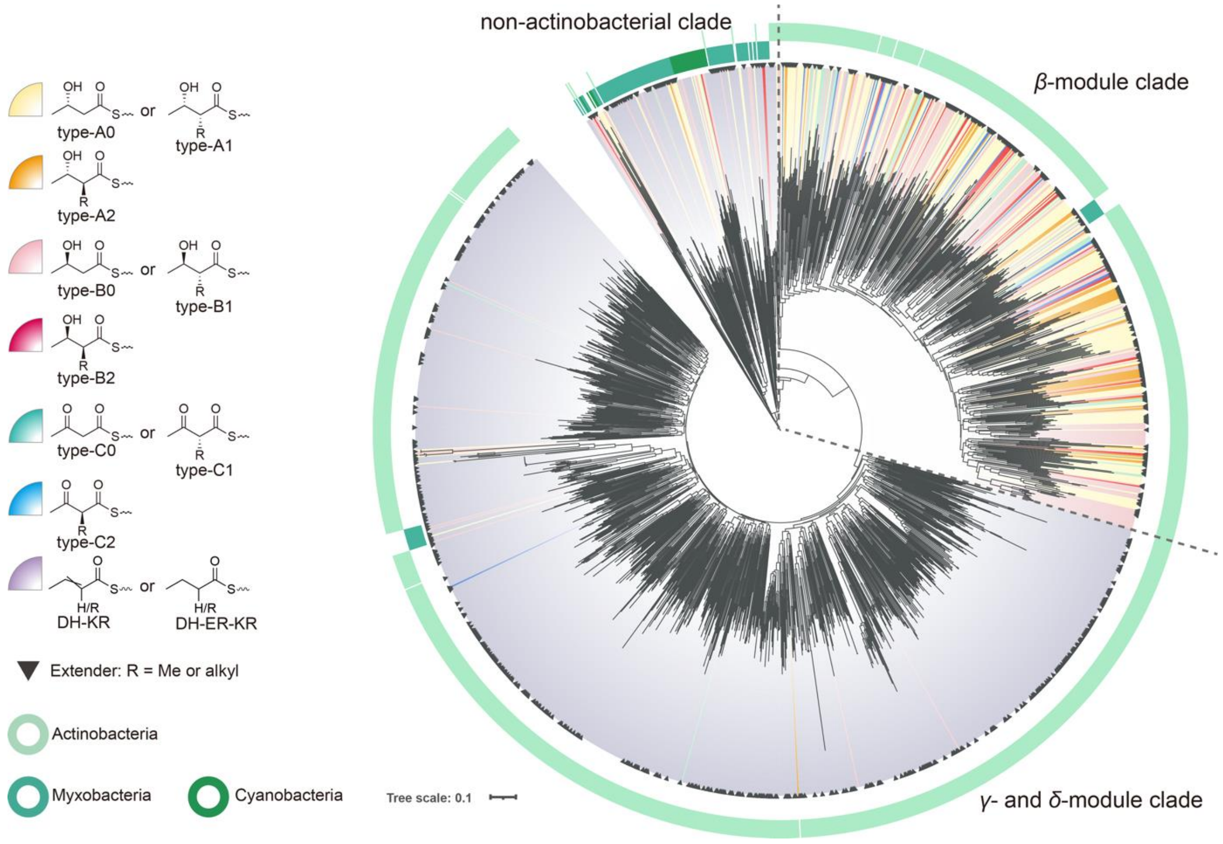

Figure S1. Phylogenetic tree of KR$_S$. The colors of sectors represent different types of KR$_S$. The colors of rings represent the species category of KR$_S$. The inverted triangle indicates that the products have α-substituents.

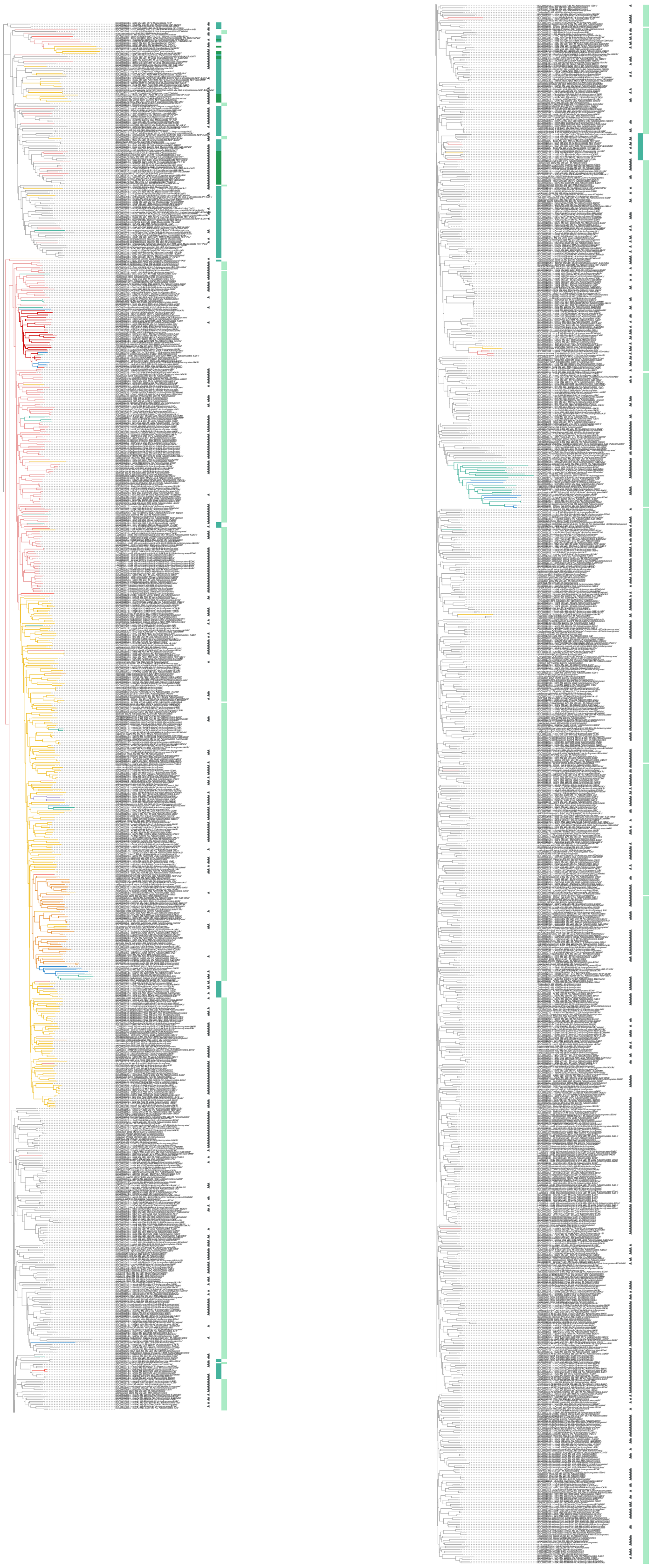

Figure S2. Phylogenetic tree of KR_C in a rectangular format. The tree is identical to Figure 2b, separated into two columns. Labels indicate the MIBiG ID or compound name, product type, taxonomy and upstream substrate information. See Supporting Information File 2 for details.

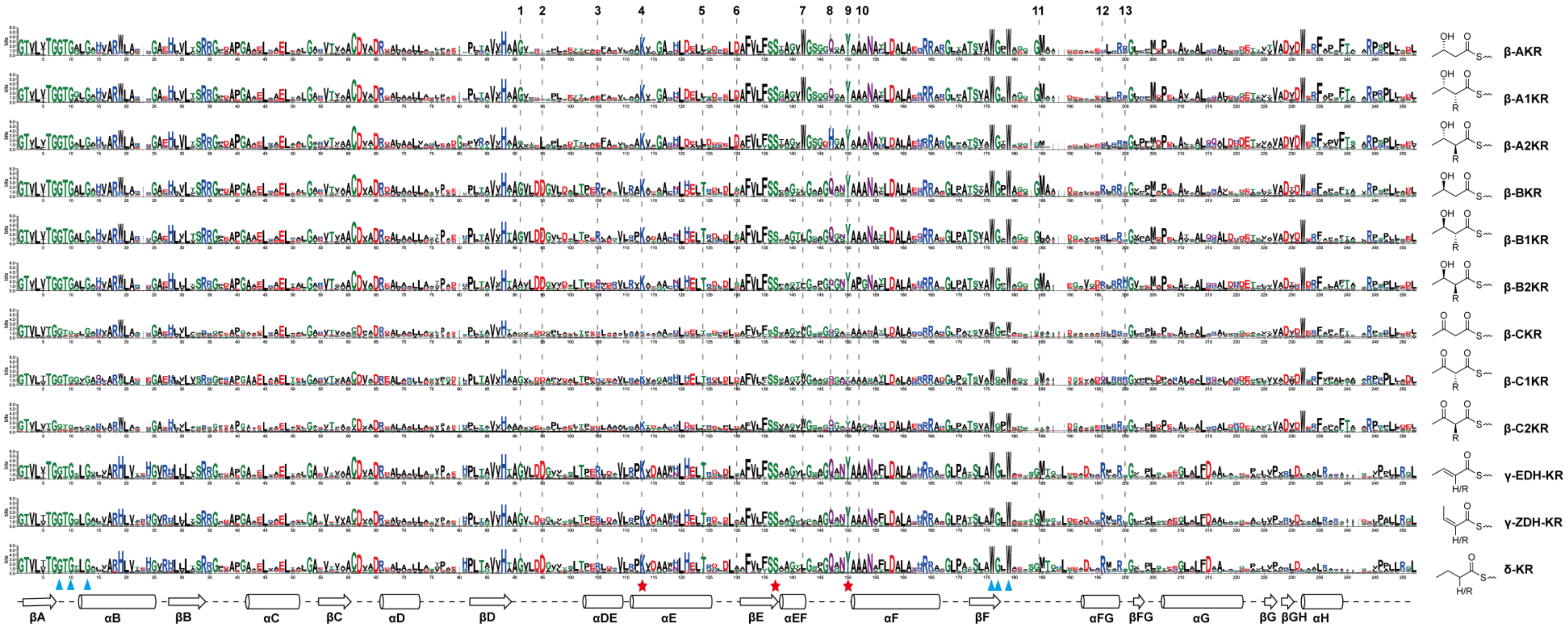

Figure S3. Sequence logo comparation of actinobacterial KR_C based on the classification of their products. The key catalytic residues are marked by red stars, and the NADPH-binding residues are marked by blue triangles.[1] The numbers at the top are used to indicate the fingerprints discussed, which correspond to those in Figure 3a.

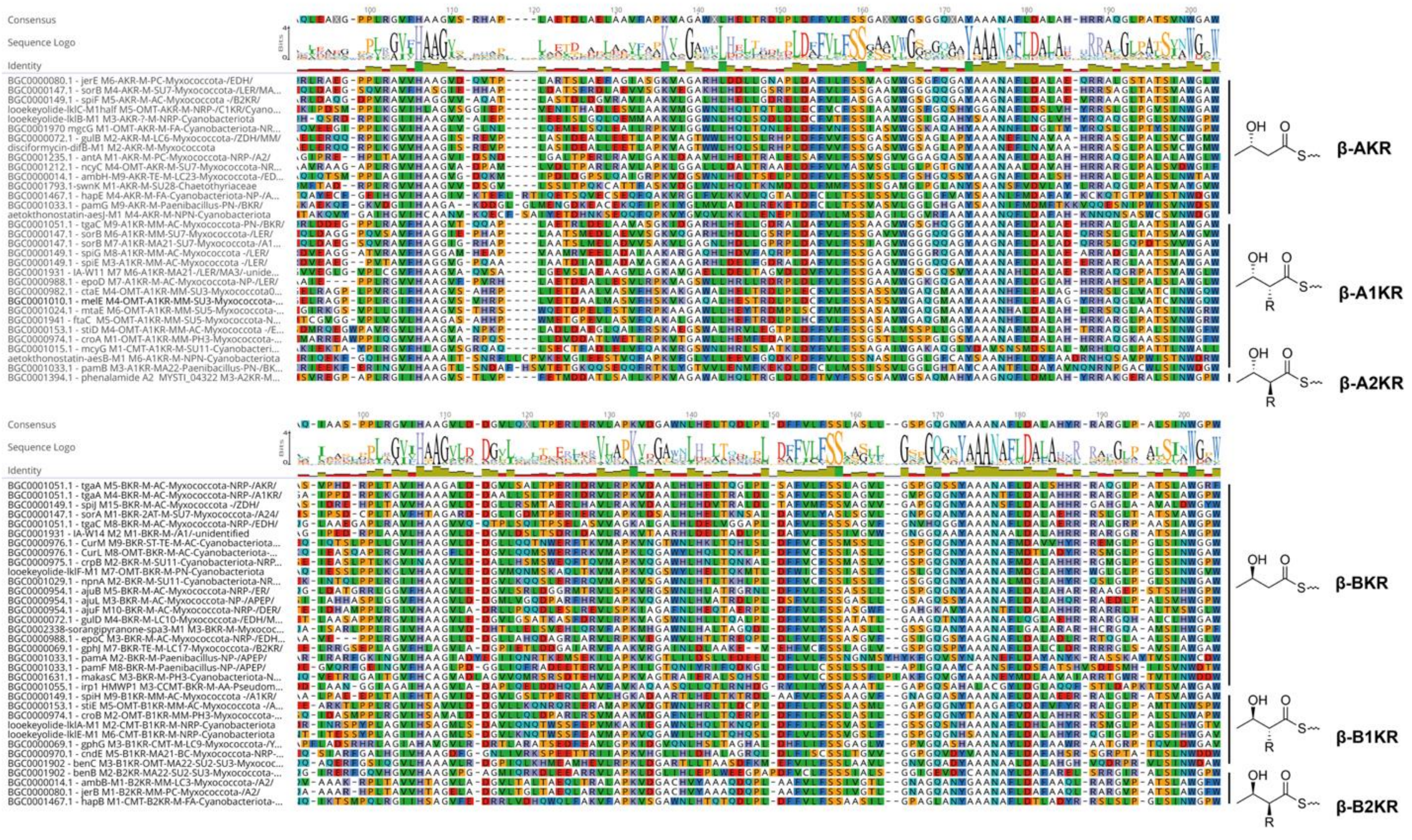

Figure S4. Sequence alignment of KR_C from non-actinobacterial β-modules. On the right are the structures of their products.

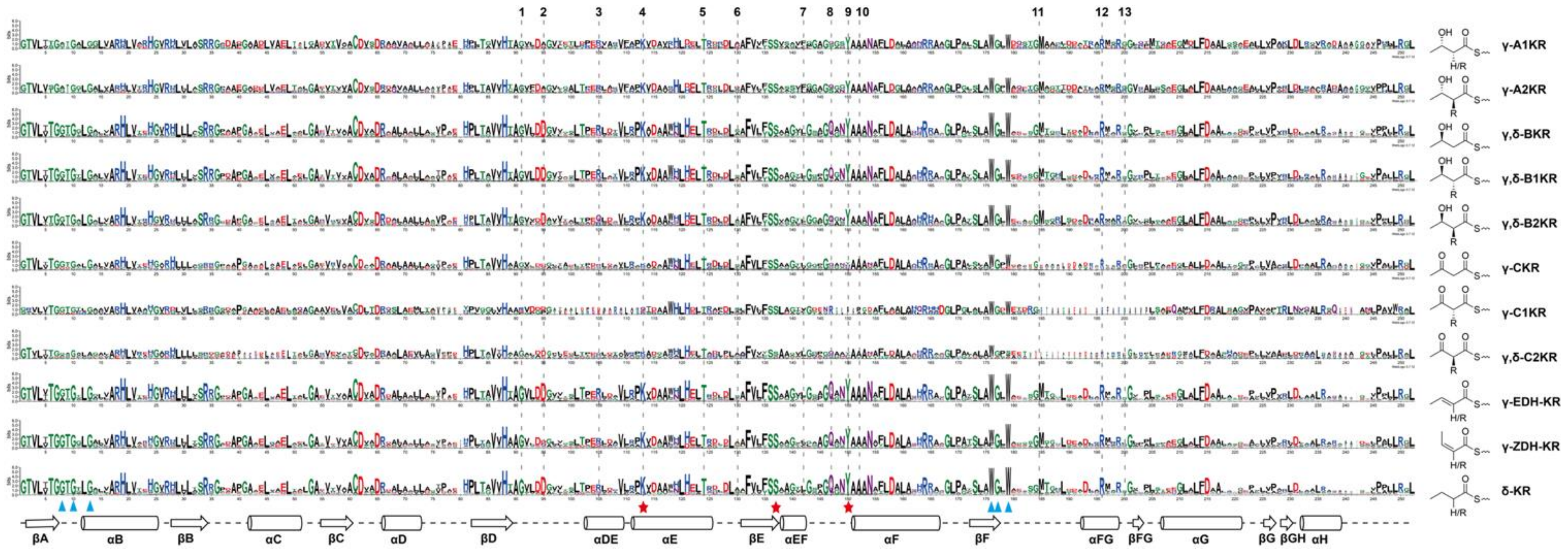

Figure S5. Sequence logo comparation of KR_C from actinobacterial γ- and δ-modules, based on the classification of their products. The key catalytic residues are marked by red stars, and the NADPH-binding residues are marked by blue triangles. The numbers at the top are used to indicate the fingerprints motifs.

	KR motifs													
	G (1)	**LDD (2)**	R/Q (3)	**K (4)**	**T (5)**	**D (6)**	**W (7)**	**H (8)**	**Y (9)**	**P (10)**	GM (11)	R (12)	H (13)	NADPH binding motifs
**A0/A1**	G	xxx	x	K	x	**D**	**W**	Q	Y	A	GM	x	x	intact
**A0/A2**	G/A	xx**L**	x	K	x	**D**	**W**	**H**	Y	A	xx	x	x	intact
**B0/B1**	G	**LDD**	R	K	**T**	x	x	Q	Y	A	GM	R	x	intact
**B0/B2**	G/A	**LDD**	R/Q	K	**T**	x	x	Q/L	Y	**P**	GM	R	H	intact
**C0/C1**	G	xxx	x	**x**	x	x	x	x	**x**	x	xx	x	x	incomplete
**C0/C2**	no G	xxx	x	K	x	x	x	Q/H	**Y/Q**	A/P	xx	x	x	incomplete

Table S1. Stereospecificity-conferring motifs summary of KRs from actinobacterial β-modules. Motifs marked in red indicate strong motifs, and others indicate supportive motifs.

	Motif (KR$_C$)											Prediction			Product
	**2**	**3**	**4**	**5**	**6**	**7**	**8**	**9**	**10**	**12**	**13**	Previous	Updated	Clade	
Caniferolide M19	**LDD**	**Q**	K	**T**	S	I	H	Y	**P**	R	H	B2	B2	B2	B1
Epemicin M13	**LDD**	**R**	K	**T**	T	W	Q	Y	A	R	G	—	B1	B1	B1
Epemicin M16	**LDD**	**R**	K	**T**	S	F	Q	Y	**P**	M	H	B2	B2	B2	B1
Filipin M1	**LDD**	**R**	K	**T**	D	W	Q	Y	A	H	R	—	B1	B1	B1
Ibomycin M7	GAE	D	K	A	D	L	R	Y	A	V	L	—	A1	A1	B1
Neaumycin M9	GQL	E	K	L	**D**	**W**	Q	Y	A	L	R	A1	A2	A1	A2
Salinomycin M9	VQT	D	K	L	**D**	**W**	**H**	Y	P	Y	R	A2	A2	A1	A1
Tylactone M1	**LDD**	S	K	**T**	D	W	Q	Y	A	S	R	—	B1	B1	B1
Ibomycin M17	**LED**	S	K	**T**	D	W	Q	Y	A	R	G	—	B1	γ/δ-	A1

Table S2. Stereochemical prediction of selected KR sequences. Motifs used for prediction are marked in bold. Prediction outcomes based on previous criteria (LDD, W, H, and P motifs) and the updated criteria, as well as based on phylogenetic cladogram, were provided.

References

1. Keatinge-Clay, A. T.; Stroud, R. M. The structure of a ketoreductase determines the organization of the beta-carbon processing enzymes of modular polyketide synthases. *Structure* **2006**, *14*, 737-748.
